# Incidental Acute ST Elevation Due to Cannabis-Induced Myocarditis After a Mechanical Fall

**DOI:** 10.1016/j.cjco.2021.05.008

**Published:** 2021-05-18

**Authors:** Melissa Tso, Dominique J. Kushneriuk, Teresa L. Bree, Shravan S. Nosib

**Affiliations:** a Department of Medicine, University of Saskatchewan, Royal University Hospital, Saskatoon, Saskatchewan, Canada; b Division of Cardiology, Department of Medicine, University of Saskatchewan, Royal University Hospital, Saskatoon, Saskatchewan, Canada

## Abstract

We present the case of a young male with an orthopedic injury after a mechanical fall, who developed atypical chest pain associated with ST elevation and elevated biomarkers suggestive of myocardial injury. He was found to have myocarditis on cardiac magnetic resonance imaging that we postulate was secondary to inhalation of marijuana. Cannabis-induced myocarditis and its potential complications are a health hazard that is bound to grow with the legalization of marijuana use in many countries.

## Résumé

Nous présentons ici le cas d'un jeune homme souffrant d'une lésion orthopédique subie après une chute mécanique, qui a développé une douleur thoracique atypique associée à une élévation du segment ST et à des biomarqueurs élevés évocateurs d'une lésion myocardique. L'imagerie par résonance magnétique cardiaque a révélé qu'il souffrait d'une myocardite que nous supposons être consécutive à l'inhalation de marijuana. La myocardite induite par le cannabis et ses complications potentielles constituent un risque pour la santé qui ne peut que croître avec la légalisation de l'usage de la marijuana dans de nombreux pays.

A 28-year-old male presented to the emergency department with an orthopedic injury 1 hour after a fall from his bike. He did not suffer chest trauma. X rays revealed a right fibular fracture that was eventually repaired. While waiting in the emergency department, he developed sudden-onset, localized, left-sided chest pain described as an "ache" within his left pectoralis muscle. There was no reproducible, positional, or pleuritic component. He had nausea but no other associated symptoms or antecedent sick contacts. He had been smoking marijuana earlier that day. He was started on management for an acute coronary syndrome, and his chest pain resolved within 1 hour. His vital sign measurements were as follows: heart rate 80-140 beats per minute; blood pressure 93/57 mm Hg; temperature 36.4°C; respiratory rate 15 breaths per minute; and oxygen saturation 100% on room air. Physical examination was unremarkable apart from right-ankle swelling and tenderness. There was no evidence of chest wall trauma. Heart sounds were normal on auscultation.

## Past Medical History

Past medical history was significant for precocious puberty, anxiety, and dyslipidemia. He smoked a pack of cigarettes and half of a marijuana joint per day. He denied intravenous drug and alcohol use. He was on chronic therapy with Sertraline.

## Differential Diagnosis

Our initial differential diagnoses included acute coronary syndrome (ie, from plaque rupture, spontaneous coronary artery dissection, coronary artery vasospasm), atypical takotsubo syndrome, and myocarditis secondary to coronavirus disease–2019 or other viral infection. Intracranial causes, cardiac contusion, and traumatic aortic dissection were unlikely given the absence of trauma to the head or chest. Early repolarization pattern was unlikely given the reciprocal electrocardiogram changes, elevated biomarkers, and symptoms.

## Investigations

An initial electrocardiogram showed ST elevation in the inferior leads (Supplemental Fig. S1, A and B) that was resolved by day 2. The high-sensitivity troponin level rose from 67.2 to 987 ng/L (< 3 ng/L). The creatine kinase level peaked at 828 (30-200 U/L). N-terminal pro hormone B-type natriuretic peptide (NT-proBNP) was 265 (< 300 ng/L). The white blood cell count was elevated at 20.79 × 109/L. His high-sensitivity C-reactive protein level was within normal range. Autoimmune and viral workups were negative, including 2 coronavirus disease–2019 swabs, human immunodeficiency virus, and hepatitis serology. A urine toxicology screen was negative for cocaine but positive for tetrahydrocannabinol (THC). D-dimer was measured at 787 (0-500 ug/L). A computed tomography pulmonary angiogram ruled out a pulmonary embolism. There were no abnormalities of the aorta.

Coronary angiography revealed normal epicardial coronary arteries (Videos 1-3 , view videos online). The left ventricular end-diastolic pressure was normal (7 mm Hg). A transthoracic echocardiogram revealed a normal left-ventricular ejection fraction > 60%, without regional wall motion abnormalities.

**Figure 1:**
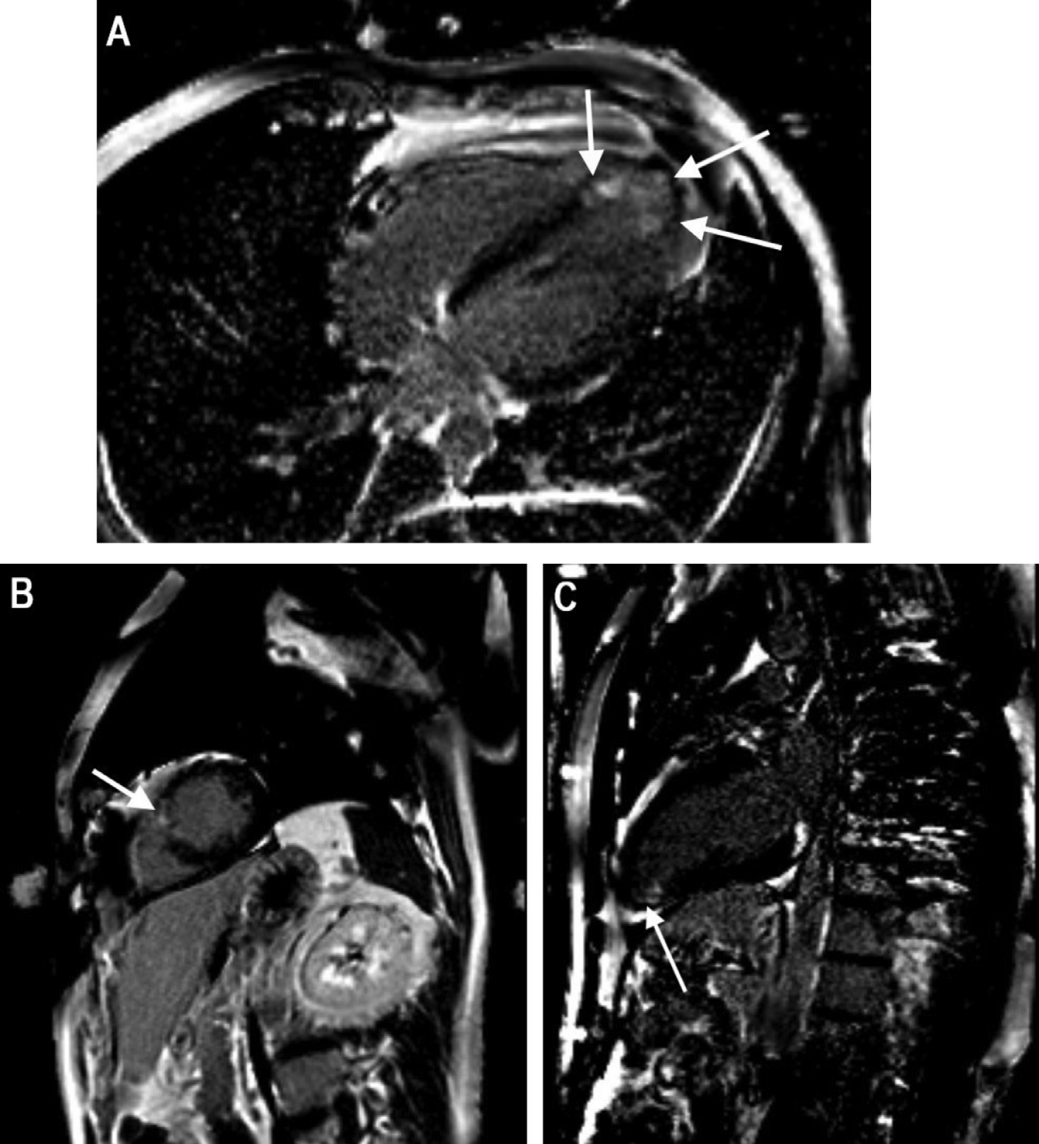
Cardiac magnetic resonance imaging shows (**arrows**) patchy mid-wall late gadolinium enhancement of the (**A, B**) left-ventricular apex, (**C**) septum, and free wall. The areas of late gadolinium enhancement are highlighted by the **white arrows**. These images (**A-C**) are phase-sensitive inversion recovery images.

Cardiac magnetic resonance imaging (MRI) revealed patchy delayed gadolinium enhancement predominantly involving the left-ventricular apex, extending into the ventricular septum and free wall (Fig. 1). The delayed enhancement appeared to be predominantly midwall in nature. There were some extensions in the septum toward the mid aspect of the left ventricle. None was demonstrated on the right. Of particular note, there was no involvement of the subendocardium of the left ventricle, the hallmark of ischemic injury, also ruling out takotsubo cardiomyopathy, as it is not typically seen in this scenario.

## Management

The typical cardiac MRI findings, in addition to the elevated cardiac biomarkers and symptoms, led to the diagnosis of myocarditis, secondary to marijuana use.

No arrhythmias were noted on continuous cardiac monitoring. Nicotine-replacement and statin were added to his therapy. He was counseled on cessation of tobacco and marijuana use. The patient was advised to refrain from high-intensity exercise for 3-6 months after discharge.

Within a week of discharge, the patient underwent an uncomplicated open reduction, internal fixation of his ankle fracture under spinal anesthetic. In outpatient review, he is asymptomatic from a cardiac perspective. He has stopped using marijuana and is concentrating on healthy living. He is back to work as a commercial truck driver.

## Discussion

Myocarditis is an inflammatory disease of the myocardium that can present in a clinical myriad, rendering it a diagnostic challenge for physicians. The proposed diagnostic criteria for clinically suspected myocarditis include ≥1 clinical presentation criterion and ≥1 diagnostic criterion from different categories. Diagnostic criteria include electrocardiogram, Holter, stress test features, elevated cardiac biomarkers, functional and structural abnormalities on cardiac imaging (transthoracic echocardiogram, angiogram, cardiac MRI), and/or tissue characterization by cardiac MRI.^1^ In asymptomatic patients, ≥2 of the diagnostic criteria are required to meet the diagnosis.

The updated Lake Louise criteria for the diagnosis of myocarditis focus on MRI evaluation of myocardial edema and injury. Increased signal intensity and relaxation time on T2-weighted imaging suggestive of myocardial edema in this case would support this diagnosis. Increased native relaxation time and extracellular volume, along with a non-ischemic pattern of late gadolinium enhancement on T1-weighted imaging would also favour a diagnosis of myocarditis. Any 2 positive findings is diagnostic of myocarditis.

It is important to note that the presence of pericardial effusion, systolic left ventricular wall motion abnormality, and high signal intensity of pericardium in late gadolinium enhancement T1, T2 mapping are supportive criteria only and not diagnostic of the same.

Endomyocardial biopsy remains the gold standard for diagnosis, although its use is limited by its invasive nature and restricted availability.

Myocarditis manifests on a wide spectrum clinically, ranging from being asymptomatic to cardiogenic shock and sudden cardiac death. Other symptoms include acute chest pain, dyspnea, fatigue, palpitations, and syncope.^2^

Our patient's case was quite unusual, as he initially presented with a noncardiac complaint, and the acuity of his chest pain was misleading. Patients that present with a myocarditis-like picture usually have symptoms that persist longer than a day. Hence, clinicians should be considering myocarditis as a differential diagnosis in any case of acute chest pain.

**Figure 2:**
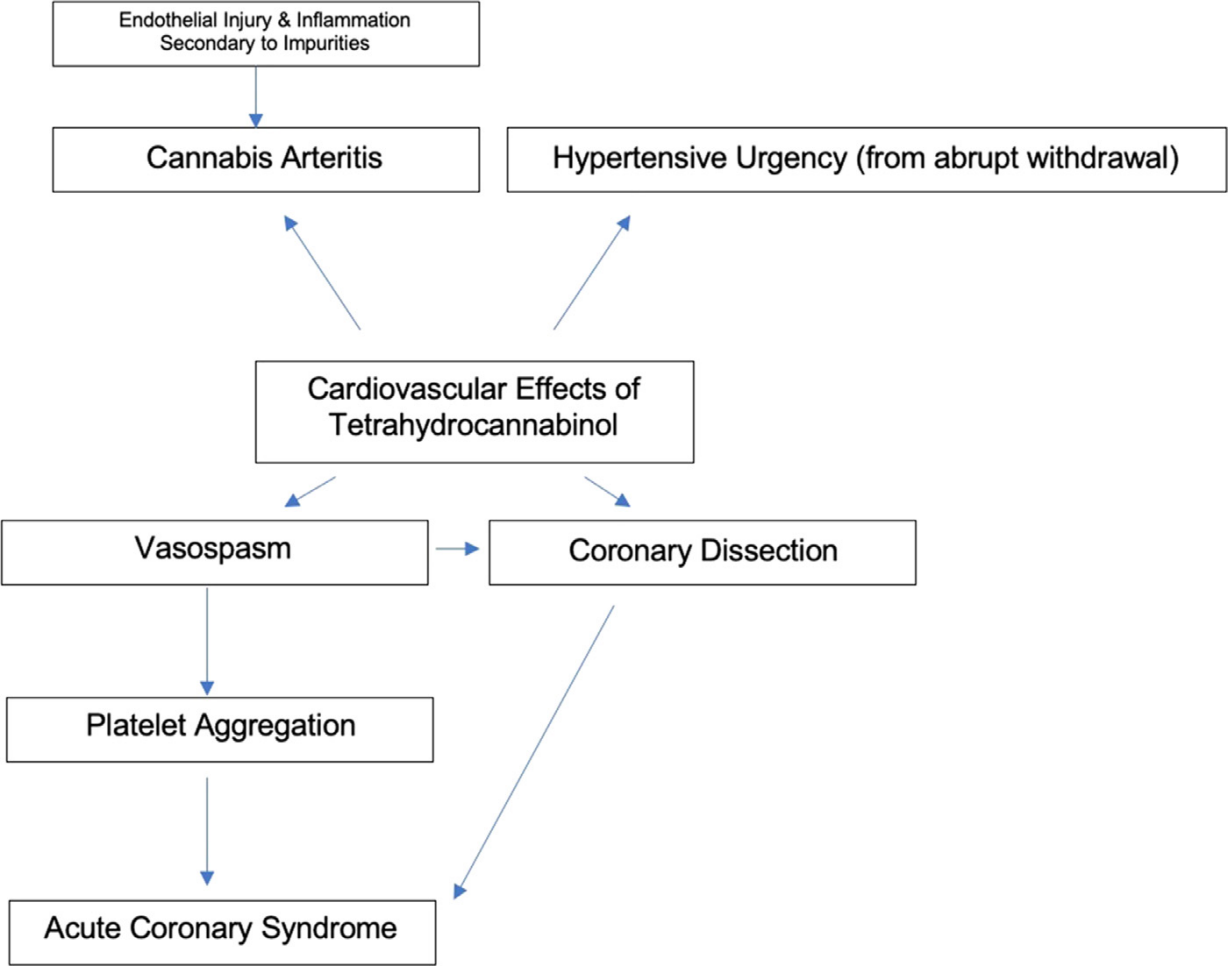
Potential cardiovascular complications of cannabis use.

Marijuana may affect the cardiovascular system through 3 different pathogenic mechanisms: marijuana-induced arteritis, coronary spasms, and platelet aggregation (Fig. 2). In fact, reversible coronary spasm is the most common cause of marijuana-induced vascular events. Coronary spasm inducing cardiomyopathy, secondary to marijuana use, has been reported on several occasions. It has been suggested that chronic marijuana users have increased susceptibility to coronary vasospasm, due to the fact that they have baseline autonomic neuropathy, which when coupled with marijuana-induced endothelial dysfunction, increases their risk of vasospasm.^3^

Although rare, there are documented case reports of marijuana being associated with myocarditis. There are endocannabinoid receptors that are located in various parts of the body, including the myocytes, and it is thought that they are involved in the regulation of heart rate and blood pressure. The pathophysiology behind the cause of myocarditis secondary to marijuana is still unclear. It has been suggested in the literature that the variety of contaminants in marijuana may play a role. In addition, cannabis has been associated with arrhythmias, acute myocardial infarctions, takotsubo cardiomyopathy, myopericarditis, and sudden cardiac death.^4^

## Conclusions

We report a rare case of marijuana-induced myocarditis. The presentation of this patient was interesting, as he initially presented with an orthopedic insult and developed cardiac symptoms in the emergency department. Although the association of cannabinoids with cardiovascular complications such as arrhythmia, sudden cardiac death, coronary artery vasospasm, and myocardial infarction has been previously established, there are only a few case reports of myocarditis. Although cannabis-induced myocarditis generally presents a benign course and good outcomes with cessation, inflammation-induced dilated cardiomyopathy may occur with continued use.^5^

The pathogenesis of marijuana-induced myocarditis/myopericarditis remains unclear. With the widespread use of cannabis, further research is needed to explore possible causes.

## Novel Teaching Points

• Myocarditis secondary to inhalation of cannabis should be considered in patients presenting with chest pain syndromes and corresponding electrical and biochemical markers of myocarditis.
• Patients should be monitored in the hospital for complications of myocarditis, such as arrhythmia.
• Counselling to encourage cessation of cannabis use is prudent.

## Funding Sources

There are no funding sources to declare.

## Disclosures

The authors have no conflicts of interest to disclose.

## Appendix Supplementary materials

Image, application 1

**Video 1.** Left ventriculogram, RAO. This shows an inferoapical wall motion abnormality. Image, video 2

**Video 2.** Right radial approach with a multi catheter, LAO 30 degrees. Right coronary artery appears normal. Image, video 3

**Video 3.** Supplemental: Right radial approach with a multi catheter, RAO 30 degrees and caudal 30 degrees. The left coronary system is normal. Image, video 4

## References

[1] Caforio A, Pankuweit S, Arbustini E (2013). Current state of knowledge on aetiology, diagnosis, management, and therapy of myocarditis: a position statement of the European Society of Cardiology Working Group on Myocardial and Pericardial Diseases. Eur Heart J.

[2] Tschöpe C, Cooper LT, Torre-Amione G, Van Linthout S. (2019). Management of myocarditis-related cardiomyopathy in adults. Circ Res.

[3] Subramaniam VN, Menezes AR, DeSchutter A (2019). The cardiovascular effects of marijuana: Are the potential adverse effects worth the high?. Mo Med.

[4] Goyal H, Awad HH, Ghali JK. (2017). Role of cannabis in cardiovascular disorders. J Thorac Dis.

[5] Kariyanna PT, Jayarangaiah A, Singh N (2018). Marijuana-induced myocarditis: a new entity of toxic myocarditis. Am J Med Case Rep.

