# Supplementary material for: Incidental Acute ST Elevation Due to Cannabis-Induced Myocarditis After a Mechanical Fall
Source: CJC Open. 2021 May 18;3(10):1303–6. doi: 10.1016/j.cjco.2021.05.008 (PMC8636242; doi:10.1016/j.cjco.2021.05.008)
Supplement: Supplementary file 1 [file mmc1.pdf]

Supplementary Material

A

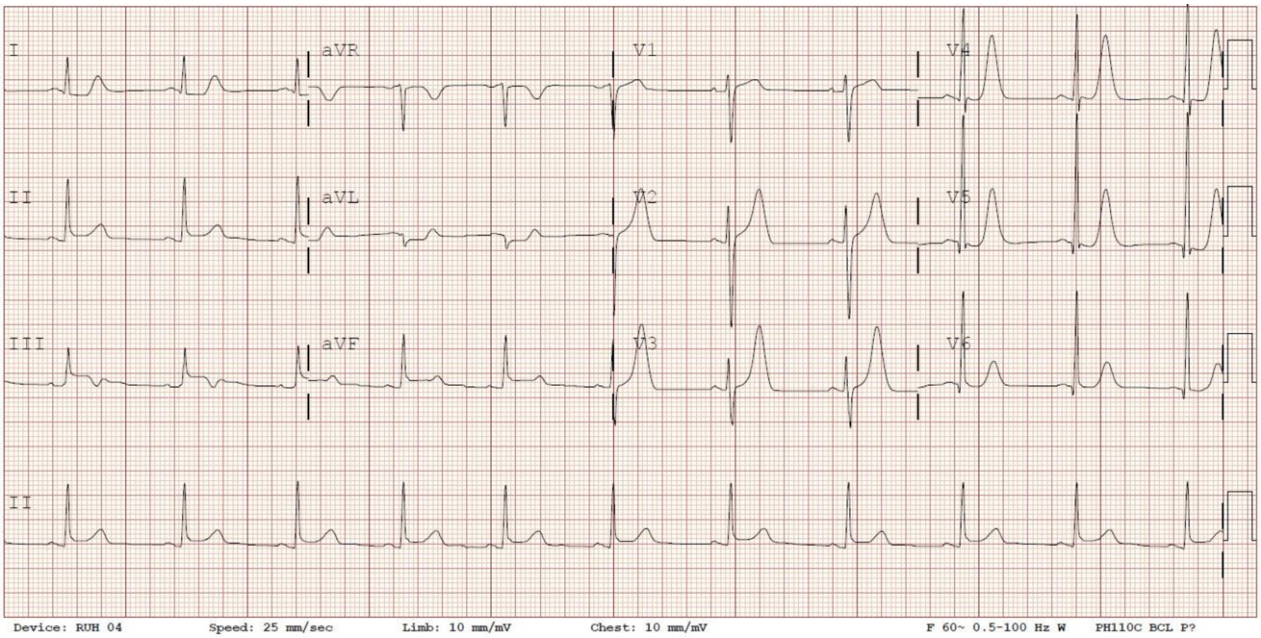

B

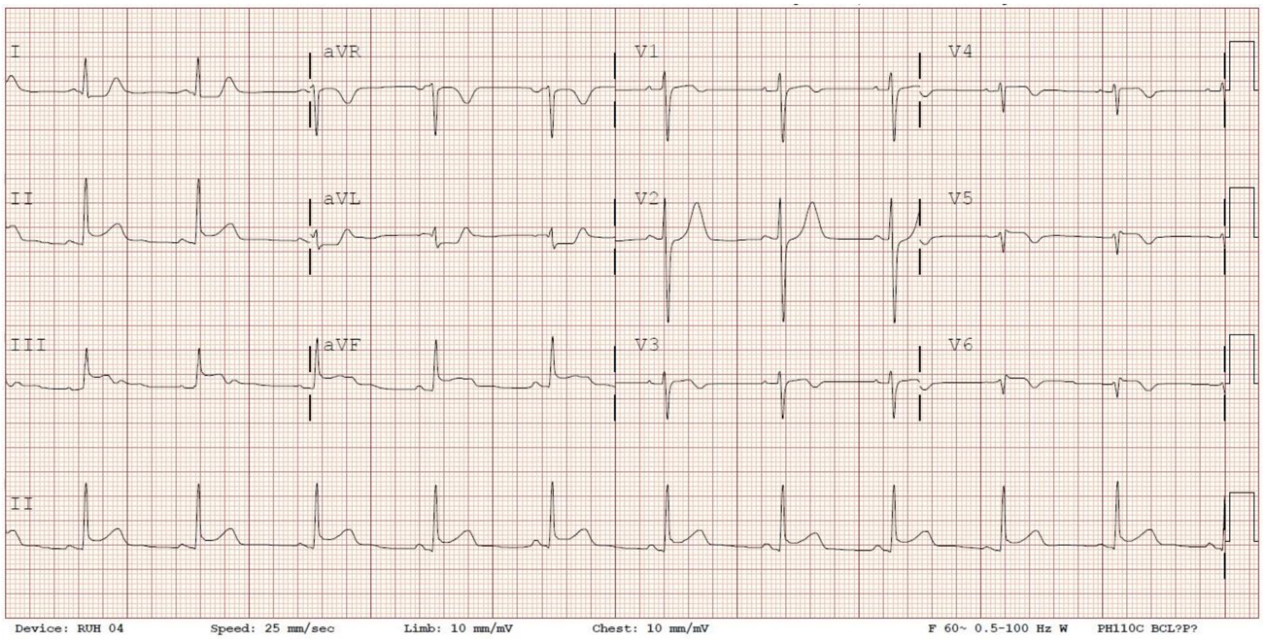

Supplemental Figure S1. (A) Initial electrocardiogram (ECG). This shows normal sinus rhythm with ST elevation in the inferior leads. There is reciprocal ST depression in the high lateral leads. **(B)** This ECG shows ST elevation in the right sided leads (V3-6).
